# Acute Kidney Injury Caused by Rhabdomyolysis Is Ameliorated by Serum Albumin-Based Supersulfide Donors through Antioxidative Pathways

**DOI:** 10.3390/ph17010128

**Published:** 2024-01-18

**Authors:** Mayumi Ikeda-Imafuku, Tatsuya Fukuta, Victor Tuan Giam Chuang, Tomohiro Sawa, Toru Maruyama, Masaki Otagiri, Tatsuhiro Ishida, Yu Ishima

**Affiliations:** 1 Department of Physical Pharmaceutics, School of Pharmaceutical Science, Wakayama Medical University, 25-1 Shichibancho, Wakayama 640-8156, Japan; imayu@wakayama-med.ac.jp (M.I.-I.); fukuta@wakayama-med.ac.jp (T.F.); 2 Department of Pharmacokinetics and Biopharmaceutics, Institute of Biomedical Sciences, Tokushima University, 1-78-1 Sho-machi, Tokushima 770-8505, Japan; ishida@tokushima-u.ac.jp; 3 Curtin Medical School, Faculty of Health Sciences, Curtin University, Perth 6845, Australia; v.chuang@curtin.edu.au; 4 Department of Microbiology, Graduate School of Medical Sciences, Kumamoto University, Kumamoto 860-8556, Japan; sawat@kumamoto-u.ac.jp; 5 Department of Biopharmaceutics, Graduate School of Pharmaceutical Sciences, Kumamoto University, 5-1 Oe-honmachi, Kumamoto 862-0973, Japan; tomaru@gpo.kumamoto-u.ac.jp; 6 Faculty of Pharmaceutical Sciences, Sojo University, 4-22-1 Ikeda, Kumamoto 860-0082, Japan; otagirim@ph.sojo-u.ac.jp; 7 Laboratory of Biopharmaceutics, Kyoto Pharmaceutical University 5 Nakauchi-cho, Misasagi, Yamashina-ku, Kyoto 607-8414, Japan

**Keywords:** supersulfides, serum albumin, acute kidney injury, oxidative stress, reactive oxygen species

## Abstract

Oxidative stress is responsible for the onset and progression of various kinds of diseases including rhabdomyolysis-induced acute kidney injury (AKI). Antioxidants are, therefore, thought to aid in the recovery of illnesses linked to oxidative stress. Supersulfide species have been shown to have substantial antioxidative activity; however, due to their limited bioavailability, few supersulfide donors have had their actions evaluated in vivo. In this study, human serum albumin (HSA) and N-acetyl-L-cysteine polysulfides (NACSn), which have polysulfides in an oxidized form, were conjugated to create a supersulfide donor. HSA is chosen to be a carrier of NACSn because of its extended blood circulation and high level of biocompatibility. In contrast to a supersulfide donor containing reduced polysulfide in HSA, the NACSn-conjugated HSAs exhibited stronger antioxidant activity than HSA and free NACSn without being uptaken by the cells in vitro. The supersulfide donor reduced the levels of blood urea nitrogen and serum creatinine significantly in a mouse model of rhabdomyolysis-induced AKI. Supersulfide donors significantly reduced the expression of oxidative stress markers in the kidney. These results indicate that the developed supersulfide donor has the therapeutic effect on rhabdomyolysis-induced AKI.

## 1. Introduction

Rhabdomyolysis is a clinical syndrome caused by the rapid disruptions of skeletal muscle and the release of myoglobin (Mb) and other intracellular components into blood circulation [1]. Trauma, ischemia, surgery, and some drugs are known to trigger rhabdomyolysis. Rhabdomyolysis can also be induced by inflammatory cytokines from viral infections, and case reports about the connection to COVID-19 have been published [2]. The most severe complication of rhabdomyolysis is acute kidney injury (AKI), which accounts for 10–50% of rhabdomyolysis cases [3]. The mortality rate of rhabdomyolysis-induced AKI patients is as high as 25–60% [4,5]. One of the main mediators of AKI by rhabdomyolysis is oxidative stress caused by Mb, a heme protein containing irons. Iron produces reactive oxygen species (ROS) such as hydroxyl radicals by Fenton reactions (Fe^2+^ + H_2_O_2_ → Fe^3+^ + ·OH) and induces apoptosis in renal tubular cells [6]. Antioxidants are, therefore, thought to treat rhabdomyolysis-induced AKI. 

Supersulfides are one of the most featured antioxidants that scavenge ROS efficiently [7,8]. Supersulfides are compounds that have catenated sulfur atoms, such as persulfides (R-SSH) and polysulfides (R-SS_n_-R′, *n* > 1). These sulfur compounds were referred to as “reactive sulfur species” in the previous decade, but Akaike et al. just defined the word “supersulfides” in 2023 [7]. The revelation that cysteine persulfide is responsible for the metabolism of electrophile 8-nitro-cGMP marked the beginning of a sharp increase in research on supersulfides [9,10]. It was found that supersulfides exist in our bodies and that there are several enzymes that can produce supersulfides [10,11,12]. The high antioxidative activity of supersulfides has been investigated and their therapeutic effect has been studied. For instance, glutathione trisulfide (GSSSG) prevented lung damage in chronic obstructive pulmonary disease model mice [13], and intranasal GSSSG improved delayed paraplegia in the spinal cord ischemia model mice [14].

Various donors of supersulfides have been developed and their activities reviewed [15]. Some functional donors release supersulfides in response to oxidative stress or prodrugs that are activated by enzymes [16,17]. However, the small molecular size of many supersulfide donors usually gets eliminated via glomerular filtration, leading to a short half-life in blood, thus, frequent dosing and high dosing amounts are needed in vivo. Therefore, formulation innovations are essential for the application of supersulfides as pharmaceuticals. There are several reports on hydrogen sulfide donors using carriers such as polymers [18] or nanoparticles [19], but not many for supersulfides. Dillon et al. developed a polymer-based persulfide prodrug, but there is no information on its in vivo activity [20].

**Figure 1:**
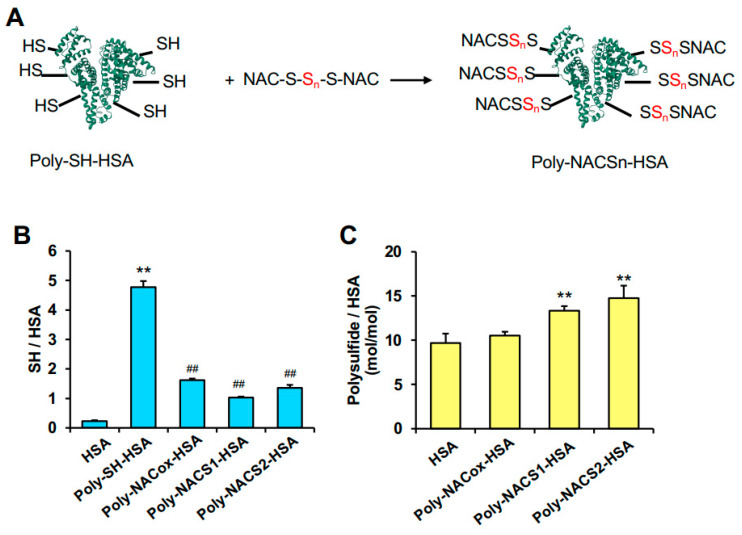
Preparation and characterization of poly-NAC-Sn-HSA. (**A**) Reaction scheme of poly-NAC-Sn-HSA. NAC polysulfides were bound to poly-SH-HSA by SH/SS exchange reactions. Red color indicates sulfane sulphur. (**B**) The levels of thiols per protein (mol/mol) were measured by DTNB assay. (**C**) The amount of polysulfides in an oxidized form was measured by EMSP assay. *n* = 3. ** *p* < 0.01 as compared with HSA. ## *p* < 0.05 as compared with Poly-SH-HSA.

Human serum albumin (HSA) is a versatile drug delivery carrier due to its high biocompatibility and long blood circulation [21]. We have previously developed supersulfide-loaded albumin (S_n_-HSA) by reacting Na_2_S_n_ with HSA and showed its antioxidant activity [22]. The reduced polysulfide in Sn-HSA exhibits instantaneous antioxidant potency. In this study, we studied the therapeutic effect of Sn-HSA on rhabdomyolysis-induced AKI mice. In addition, we have focused on NAC polysulfide, a supersulfide donor in the spotlight, which has an oxidized polysulfide in N-acetyl-L-cystine. Since N-acetyl-L-cysteine has a short blood circulation half-life of within one hour [23], we attempted to prolong the blood circulation half-life of NAC polysulfide by conjugating NAC polysulfide to HSA to avoid glomerular filtration. As shown in the conjugation scheme in Figure 1A, NAC polysulfides were introduced to HSA by SS/SH exchange reactions, resulting in the induction of an oxidized polysulfide in HSA. It is not entirely clear how the reduced and oxidized forms of supersulfide differ in their therapeutic effects on oxidative stress illnesses. In this study, we evaluated the therapeutic efficacy of two different types of supersulfide donors in their oxidized and reduced forms using HSA as a carrier in rhabdomyolysis-induced AKI mice.

## 2. Results

### 2.1. Development of a Novel Supersulfide-Bound Serum Albumin

We have previously reported that Na_2_S_n_ treatment provides reduced polysulfides to HSA [22]. Na_2_S_n_-treated HSA showed stronger antioxidant activities and suppressed melanin synthesis. Another study also suggested that NaHS-treatment induces hydropersulfide in possibly Cys34 of HSA. Here, we tried to develop a new supersulfide donor that has oxidized polysulfides in HSA using NAC polysulfides, a small molecule that have polysulfides in an oxidized form reported by Zhang et al. [24]. The reaction scheme is illustrated in Figure 1A. Thiols were induced to serum albumin using Traut’s reagent, as reported previously, for the synthesis of poly-nitrosylated serum albumin [25]. Reaction conditions with Traut’s reagent were the same as Katayama et al. [25]. Following the reaction between thiol-induced HSA (poly-SH-HSA) and NAC polysulfides, it is anticipated that the NAC polysulfides would bind to poly-SH-HSA by SH/SS exchange reactions. As NAC polysulfides, NAC-S1 and NAC-S2, which include one and two sulfane sulphur in disulphide linkages, respectively, were produced as NAC polysulfides [24]. Poly-SH-HSA reacted with NAC-S1 and NAC-S2 to form poly-NAC-S1-HSA and poly-NAC-S2-HSA, respectively, whereas poly-SH-HSA reacted with NAC disulfide to form poly-NACox-HSA.

To characterize poly-NACSn-HSA, the levels of thiol were measured using the DTNB method. About five thiols per HSA were detected, and three or four of them are oxidized by the reactions with NAC polysulfides or NAC disulfide (Figure 1B). The number of polysulfides in oxidized forms was analyzed by following our previous report [26]. Poly-NAC-S1-HSA and poly-NAC-S2-HSA had about four and five more polysulfides than plain HSA, while there was no significant difference between HSA and poly-NACox-HSA (Figure 1C), suggesting that polysulfides were provided to HSA from NAC polysulfides.

### 2.2. Intracellular Uptake of Supersulfides and Antioxidative Activity

**Figure 2:**
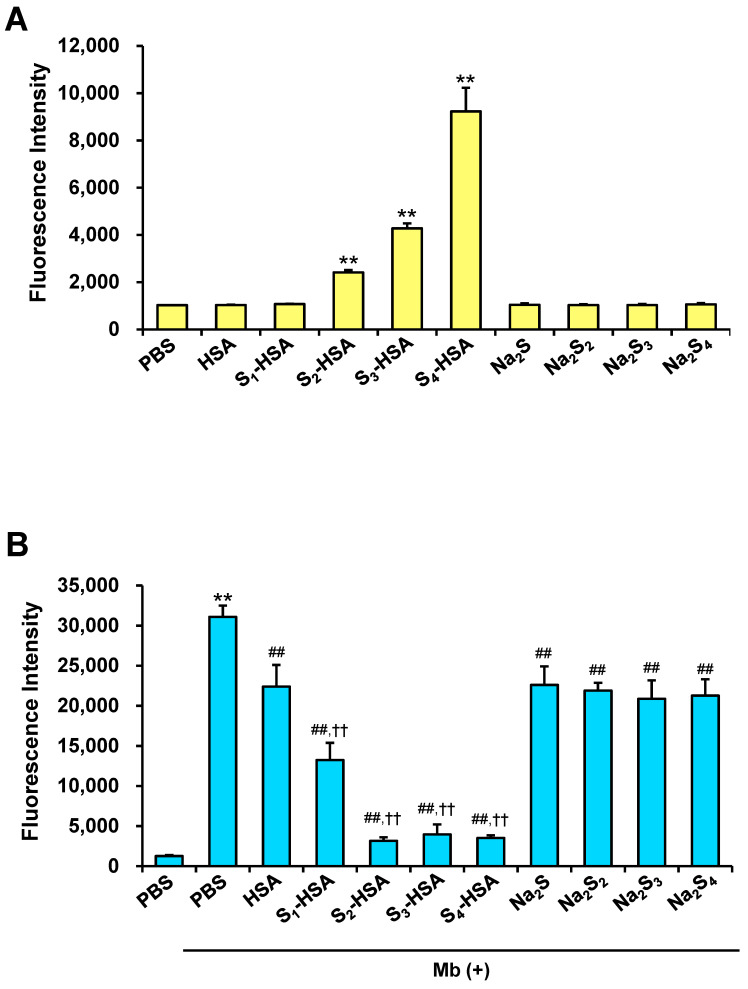
Supersulfide supply and antioxidant activity of S_n_-HSA in vitro. (**A**) Cellular uptake of supersulfides detected by a supersulfides fluorescent probe, SSP4, in LLC-PK1 cells. Samples (20 μM) were incubated with the cells for 2 h and washed with PBS, followed by SSP4 (5 μM) treatment. ** *p* < 0.01 as compared with PBS. (**B**) The fluorescence intensity of CM-H_2_DCF-DA in LLC-PK1 cells for measuring ROS induced by myoglobin (Mb). Samples (10 μM) were treated with the cells for 2 h with Mb (0.1 mg/mL) after the pretreatment of CM-H_2_DCF-DA (5 μM). ** *p* < 0.01 as compared with PBS without Mb. ## *p* < 0.01 as compared with PBS plus Mb. ^††^ *p* < 0.01 as compared with HSA.

The antioxidative activities of S_n_-HSA to ROS induced by ultraviolet in mouse melanoma cells were studied in a previous study [22]. In this study, we evaluated the antioxidant effect of S_n_-HSA on ROS induced by myoglobin in kidney cells to assess whether S_n_-HSA has the potential to treat rhabdomyolysis-induced AKI. Sulfane sulfur probe 4 (SSP4), a fluorescent probe for polysulfides, was used in advance to evaluate the cellular uptake of S_n_-HSA in pig kidney epithelial cells, LLC-PK1 cells (Figure 2A). After giving the cells two hours of treatment with S_n_-HSA or Na_2_S_n_ (20 μM), sufficient washes were performed before applying the SSP4 solution in a positive-charged surfactant. The mean fluorescence intensity was significantly higher in the S_2_-HSA, S_3_-HSA, and S_4_-HSA groups compared to phosphate-buffered saline (PBS), while there was no change in the Na_2_S_n_ groups. CM-H_2_DCF-DA pretreatment was performed before detecting ROS induced by Mb in LLC-PK1 cells. After the removal of CM-H_2_DCF-DA, Mb and the samples (10 μM) were added to the cells and the fluorescence intensity was measured. The levels of ROS increased by Mb and S_n_-HSA significantly decreased them (Figure 2B). Although HSA and Na_2_S_n_ suppressed ROS, the antioxidant effect of S_n_-HSA was remarkably higher. 

**Figure 3:**
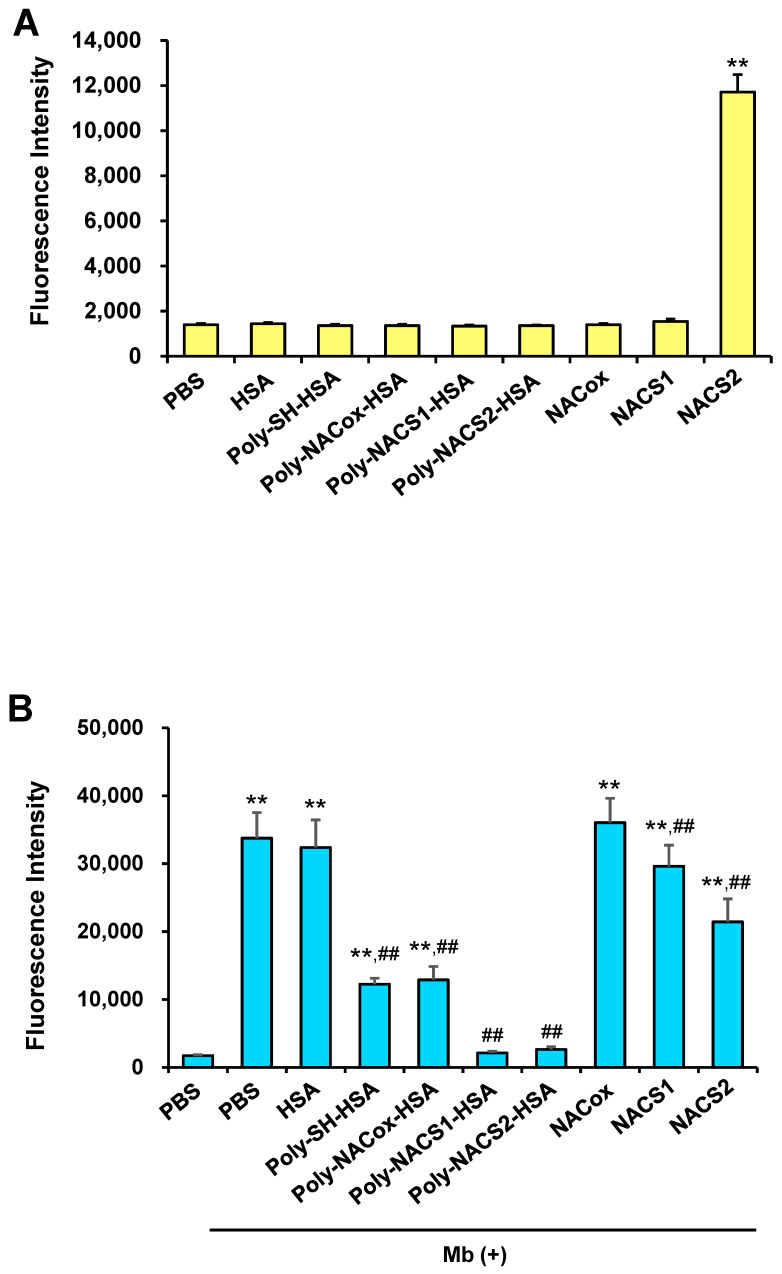
Supersulfides supply and antioxidant activity of poly-NAC-Sn-HSA in vitro. (**A**) Cellular uptake of supersulfides detected by SSP4. Samples (20 μM) were incubated with the cells for 2 h and washed with PBS, followed by SSP4 (5 μM) treatment. ** *p* < 0.01 as compared with PBS. (**B**) The fluorescence intensity of CM-H_2_DCF-DA in LLC-PK1 cells for measuring ROS induced by Mb. Poly-NACSn-HSA (10 μM) and NAC polysulfides (50 μM) were treated with the cells for 2 h with Mb (0.1 mg/mL) after the pretreatment of CM-H_2_DCF-DA (5 μM). ** *p* < 0.01 as compared with PBS without Mb. ## *p* < 0.01 as compared with PBS plus Mb. *n* = 3.

Likewise, the antioxidant activity and cellular supersulfides transport effect of poly-NAC-Sn-HSA were evaluated using the same methods. The cellular levels of supersulfides detected by SSP4 were not altered by Poly-NAC-Sn-HSA, whereas NAC-S2 increased significantly (Figure 3A). Therefore, the antioxidant effect of poly-NAC-Sn-HSA was evaluated by measuring its suppression of myoglobin-induced oxidative stress. The outcomes showed that ROS were nearly completely suppressed by poly-NAC-S1-HSA and poly-NAC-S2-HSA (Figure 3B). Poly-NAC-S1-HSA and poly-NAC-S2-HSA showed higher antioxidant activity than poly-NACox-HSA at the same concentration. This suggests that at least part of their antioxidant activity is mediated by polysulfides. Furthermore, these antioxidant activities were five times higher than NAC polysulfide or much higher than the same quantity of HSA, indicating that they might have acquired synergistic antioxidant effects in combination with HSA. When combined with the findings in Figure 3A,B, poly-NAC-Sn-HSA is predicted to eliminate ROS extracellularly.

### 2.3. Effect of Supersulfide Donors on Glycerol-Induced AKI

**Figure 4:**
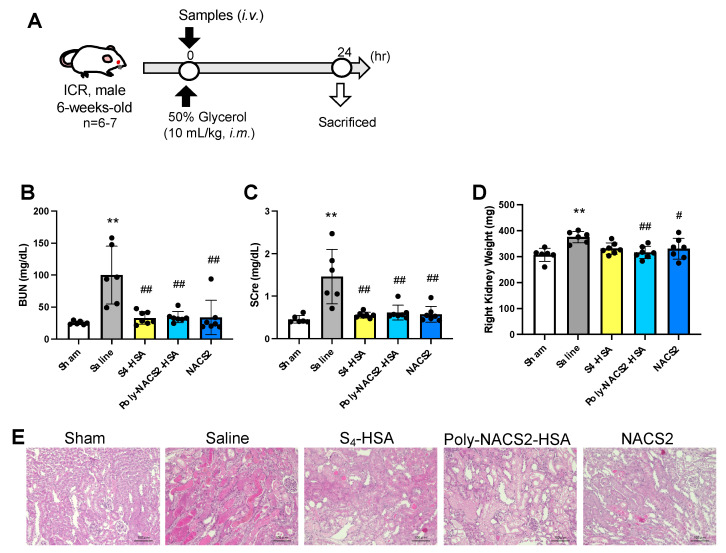
Treatment of glycerol-induced AKI by supersulfide donors. Glycerol solution (50%, 10 mL/kg) was intramuscularly injected to induce rhabdomyolysis. PBS was used instead of glycerol for the sham group. S4-HSA (1 μmol/kg), poly-NAC-S2-HSA (1 μmol/kg), and NAC-S2 (100 μmol/kg) were intravenously injected immediately after the treatment of glycerol solutions. (**A**) A model and schedule for rhabdomyolysis-induced AKI. The levels of BUN (**B**) and serum creatinine (**C**) in AKI mice 24 h after the administration of glycerol and supersulfide donors. (**D**) The weight of right kidneys. Each value represents the mean ± S.D. *n* = 6–7. ** *p* < 0.01 as compared with sham. # *p* < 0.05, ## *p* < 0.01 as compared with saline. (**E**) H.E. stains in renal tissue. The scale bar is 100 μm.

The therapeutic effect of supersulfide donors, S_4_-HSA, poly-NAC-S2-HSA, and NAC-S2 was assessed in the rhabdomyolysis-induced AKI mice model. Glycerol (50% in water) was injected into mice muscle to induce rhabdomyolysis, and supersulfide donors were administrated intravenously (Figure 4A). In comparison to S_4_-HSA and poly-NAC-S2-HSA, NAC-S2 was injected with a 100 times higher amount in order to account for its shortened blood circulation time. Serum and urine were collected 24 h after glycerol administration, and the kidneys were harvested after blood perfusion. Blood urea nitrogen (BUN), serum creatinine, and kidney weight were measured as markers of renal damage. The results showed that while AKI induction caused an approximately four-fold rise in BUN, the supersulfide donor reduced it to nearly healthy normal levels (Figure 4B). Similarly, the administration of supersulfides resulted in the suppression of serum creatinine levels to a sham level (Figure 4C). The weight of the kidneys are known to increase after being damaged by glycerol [27], and we have weighed the right kidneys. The result showed that the right kidney weight was elevated in the saline group and significantly decreased in the poly-NAC-S2-HSA and NAC-S2 groups. Furthermore, S_4_-HSA also tends to decrease the weight (*p* = 0.0524).

**Figure 5:**
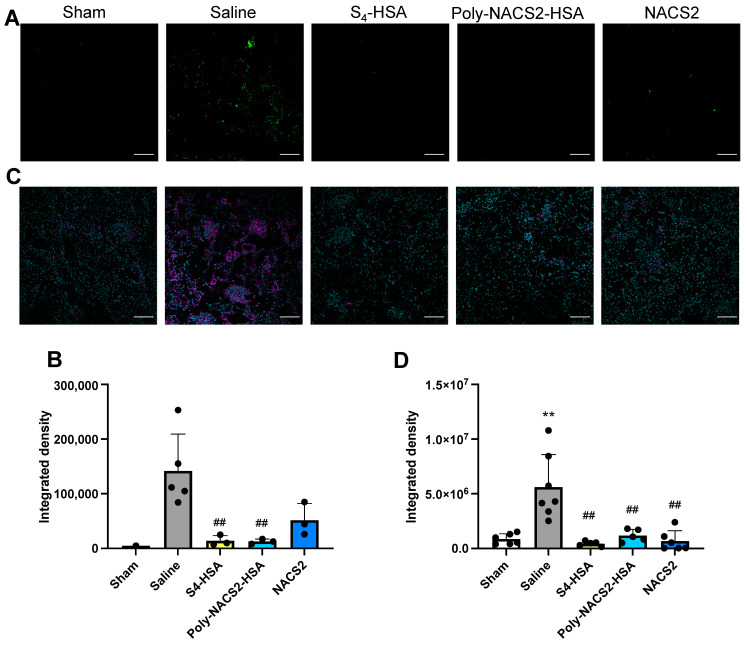
Oxidative stress and apoptosis on glycerol-induced AKI model. (**A**) TUNEL stain in kidneys to detect apoptosis. (**B**) The quantitative analysis of TUNEL stain. The scale bar is 100 μm. (**C**) Immunohistochemistry of 8-OHdG shown in magenta for the detection of oxidative stress. DAPI for nuclear stain is shown in cyan. The scale bar is 100 μm. (**D**) The quantitative analysis of immunohistochemistry of 8-OHdG in kidney tissue. ** *p* < 0.01 as compared with sham. ## *p* < 0.01 as compared with saline.

To analyze the morphological change in renal tissue, hematoxylin and eosin (H.E.) stains were performed, and severe tissue damage, such as cast formation, was observed in the saline group (Figure 4E). To further investigate renal damage, a TUNEL stain for apoptosis detection was performed. The numbers of TUNEL-positive cells stained in green were markedly increased by the glycerol treatment, while few apoptotic cells were observed in the S_4_-HSA or poly-NAC-S2-HSA-treated groups (Figure 5A). The quantitative analysis indicated a significant decrease in S_4_-HSA or poly-NAC-S2-HSA-treated groups compared to the saline group (Figure 5B). Though several TUNEL-positive cells were detected in the NAC-S2 group, the numbers were lower than that of the saline group. Immunohistochemistry for 8-hydroxy-2-deoxyguanosine (8-OHdG), which is produced when ROS damaged DNA, was executed to analyze oxidative stress in kidneys. The expression of 8-OHdG colored in magenta was confirmed in the saline group, especially around the glomerulus. In contrast, the supersulfide-treated groups showed significantly decreased levels of 8-OHdG (Figure 5C,D). 

## 3. Discussion

Oxidative stress is intimately associated with the progression of acute or chronic renal diseases [28,29]. Kidney damage is caused by apoptosis brought on by excessive oxidative stress [30]. Blood levels of uremic toxins rise and contribute to further oxidative stress when renal failure worsens and excretory function deteriorates [31]. One could argue that the key to breaking such a negative cycle is to reduce oxidative stress. Furthermore, ROS has a role in several organ illnesses, such as cardiovascular disease brought on by renal failure [29]. Intending to utilize the antioxidative impact, several groups have investigated the potential therapeutic benefits of hydrogen sulfide or supersulfides for kidney diseases [32,33]. Askari et al. demonstrated that NaHS, as a hydrogen sulfide donor, attenuated kidney dysfunctions in 5/6-nephrectomy CKD model mice by modulating redox balance [34]. Cao et al. have shown the therapeutic effect of Na_2_S_4_ on cisplatin-induced acute kidney injury [35]. The same group also reported that Na_2_S_4_ prevents diabetic nephropathy by attenuating apoptosis via NF-κB and STAT3 inactivation [36]. All of the models suggest that sulfur compounds can be used to treat kidney diseases, although the models are different, and the mechanisms are not limited to antioxidant capacity.

S_n_-HSA and poly-NAC-Sn-HSA may be more blood-retentive than current supersulfide donors due to their molecular size, which is not affected by glomerular filtration. The therapeutic efficacy of NAC-S2-HSA and S_4_-HSA was on par with or superior to 100-fold greater dosages of NAC-S2. Further investigation on the effects of supersulfide donors on blood circulation would be required. Although the effect of kidney disorders on endogenous supersulfides is poorly understood, sepsis-induced AKI patients have been shown to have lower plasma hydrogen sulfide levels [37]. Since the levels of 3-MST, one of the supersulfide-producing enzymes [12], in the kidney tissues decreased in LPS-induced AKI mice, endogenous supersulfide levels might decrease as well. Treatment with supersulfide donors might also be beneficial to restore endogenous supersulfides. Previous studies have shown that serum albumin contains supersulfides [26,38,39], and albumin-based supersulfide donors such as S_n_-HSA and poly-NAC-S2-HSA may be better since they are more biocompatible.

In this study, we developed novel supersulfide donors, poly-NAC-Sn-HSA, and assessed their therapeutic effect on glycerol-induced AKI, which is known as the clinically closest mice model of rhabdomyolysis-induced kidney failure [40]. Polysulfides were confirmed to be present in an oxidized form of Poly-NAC-Sn-HSA (Figure 1B,C). Previously, we developed another HSA-based supersulfide donor, Sn-HSA, and demonstrated their antioxidant effects [22]. S_n_-HSA is simply produced by a co-incubation with HSA and Na_2_S_n_, and S_n_-HSA has polysulfides in a reduced form. Both poly-NAC-Sn-HSA and S_n_-HSA have shown an antioxidative effect on Mb-induced ROS in vitro (Figure 2B and Figure 3B). However, there are differences between Sn-HSA and poly-NAC-Sn-HSA in the efficiency of intracellular supersulfide supplementation. The efficiency was dependent on the number of supersulfides added to HSA. The levels of supersulfides significantly increased after the S_n_-HSA treatment, whereas the number of cellular supersulfides after poly-NAC-Sn-HSA treatment remained the same, although free NAC-S2 increased. The SSP4 probe reacts with polysulfides in two different states: an oxidized form, such as S_8,_ and a reduced form, including Na_2_S_2_ [41,42]. SSP4 also reacts with persulfidated proteins, including serum albumin [41]. Therefore, the difference in cellular supersulfides after S_n_-HSA and poly-NAC-Sn-HSA treatments can be considered due to the difference in the cellular uptakes. Since both scavenged ROS in vitro, it is hypothesized that S_n_-HSA displays intracellular antioxidant activity and poly-NAC-Sn-HSA has extracellular antioxidant activity.

We have previously developed mono-SNO-HSA and poly-SNO-HSA, whereby one or multiple nitric oxides (NO) are bound per albumin, respectively, by inducing thiols with Traut’s reagent as in the synthesis of poly-NAC-Sn-HSA [25,43,44]. While both mono-SNO-HSA and poly-SNO-HSA are serum albumin-based NO carriers, their cellular uptake mechanisms are different [45]. Poly-SNO-HSA transfers NO via cellular surface thiol protein, such as protein disulfide isomerase (PDI). PDI is well known as an endoplasmic reticulum protein, but it also expresses on the cell surface in some conditions, such as cancer, and aids in the intracellular transfer of NO [45,46]. On the other hand, PDI inhibitors did not affect the cellular uptake of NO by mono-SNO-HSA, indicating that the NO transfer mechanism of mono-SNO-HSA is independent of PDI protein. Although the exact mechanism of mono-SNO-HSA transfers NO into cells is still unknown, it is partly mediated by the L-amino acid transporter. Interestingly, these differences in cellular uptake mechanisms were directly related to the differences in NO-mediated pharmacological effects [45].

As described above, there have been cases in the past where, even when the same HSA was used as the carrier, the cellular uptake differed depending on the number of binding molecules and modification methods. The LLC-PK1 cells employed in this study cannot be compared to the SNO example because they are not cancer cells; nonetheless, they would be a good candidate to investigate the mechanism of Sn-HSA uptake from a pathway comparable to the L-amino acid transporter in the future. Another possible uptake route is the transport pathway of serum albumin. Megalin and cubilin mediate the uptake of serum albumin via endocytosis in renal proximal tubule cells [47,48]. Since megalin and cubilin are expressed in LLC-PK1 cells [49,50], it is hypothesized that S_n_-HSA transferred supersulfides via megalin or cubilin in this study. However, megalin and cubilin might not recognize poly-NAC-Sn-HSA as serum albumin because more than five lysine residues of HSA are modified. Further investigation is required to shed light on the cellular uptake mechanism difference.

In rhabdomyolysis-induced model mice, both S_4_-HSA and poly-NAC-S2-HSA prevented kidney injury. The levels of BUN and serum creatinine are brought down to nearly the levels seen in healthy controls (Figure 4B,C), and neither apoptotic cells nor the expression of 8-OHdG are observed in the S_4_-HSA- and poly-NAC-S2-HSA-treated groups (Figure 5C,D). This model is severe with significant morphological changes (Figure 4E), and it is valuable to demonstrate a therapeutic effect in this model. When a traumatic injury or other event causes muscle damage in rhabdomyolysis, Mb is released from the muscle cells [51]. Since Mb is an iron-containing hemoprotein, Mb produces ROS in kidney tubular cells by the Fenton reaction. Treatment for this condition, therefore, closely correlates with lowering oxidative stress in renal tissue [52]. The remarkable suppression of 8-OHdG expression in this study provides proof that supersulfide donors suppressed oxidative stress.

There is a lot of research on the antioxidant mechanism of reduced supersulfides. Since the p*K*a of persulfides is lower than that of thiol compounds by α effect [53,54], supersulfides in a reduced form exist as anions in neutral pH and can react with oxidants directly and effectively. The mechanism by which supersulfides in an oxidized form scavenge oxidative stress has not been extensively studied, but homolytic substitution (X· + R-S-S_n_-R′ → R-SX + R′S_n_·) is proposed [55,56]. Because Mb oxidation produces radicals, poly-NAC-Sn-HSA exhibited antioxidant activity, probably via homolytic substitution. In addition, hydropersulfides are reported to reduce Fe^3+^ on myoglobin and oxymyoglobin via R′S_n_· [57]. Although little is known about the role of Fe^3+^ and oxymyoglobin on the progress of rhabdomyolysis-induced AKI, this might be related to the therapeutic of Sn-HSA and poly-NACSn-HSA since both Fe^3+^ and oxymyoglobin are oxidants.

Supersulfides have direct effects on proteins in addition to their antioxidant action, which is one of its therapeutic mechanisms. Supersulfides or hydrogen sulfide are the cause of *S*-sulfhydration, which is the conversion of thiol to persulfide [58]. *S*-sulfhydration of NF-κB at Cys-38 attenuates the activity of NF-κB and suppresses inflammation [59]. Keap-1 releases Nrf2 and promotes nuclear translocation by *S*-sulfhydration [60]. Parkin is physiologically *S*-sulfhydrated and exhibits enhanced neuroprotective activity [61]. It is interesting to note that patients with Parkinson’s disease had reduced amounts of *S*-sulfhydrated parkin, and supersulfide or hydrogen sulfide may have therapeutic uses [61]. Supersulfides have been shown in numerous instances to control protein activities through thiol modifications [62,63]. In addition, there are also cases where supersulfides bind to toxins and inactivate them. For example, when supersulfides react with methyl mercury (MeHg), they form the bis form (MeHg)_2_S and detoxicate MeHg [64]. S_n_-HSA and poly-NAC-S2-HSA are expected to exhibit a variety of actions as supersulfide donors besides their antioxidant effects.

## 4. Materials and Methods

### 4.1. Materials

Sodium polysulfides (Na_2_S_n_) were purchased from the DOJINDO chemical laboratory, Japan. Human albumin 25% “BENESIS” was provided by the Japan Blood Products Organization (Tokyo, Japan). Spectra pore 7 (MWCO: 3500) as a sulfur-free dialyzed membrane, was purchased from Repligen, USA. Ascorbic acid, dithiothreitol (DTT), zinc acetate dihydrate, LabAssay™ Creatinine, and linoleic acid were purchased from FUJIFILM Wako Pure Chemical Corporation, Osaka, Japan. The BCA protein assay kit and BUN Colorimetric Detection Kit were obtained from Thermo Fisher Scientific, Waltham, MA, USA. Other chemicals were of the best grades commercially available, and all solutions were made using deionized water.

### 4.2. Preparation of S_n_-HSA

S_n_-HSA was synthesized following our previous report [22]. In brief, defatted human serum albumin (20 mg/mL) was reacted with 1 mM of Na_2_S_n_ in PBS at 37 °C for 1 h. S_n_-HSA were then purified by using a gel-filtration column, HiTrap Desalting column (Cytiva, Marlborough, MA, USA).

### 4.3. Preparation of Poly-NACSn-HSA

HSA (150 μM) was reacted with 3 mM 2-imonothiolane (Traut’s reagent, FUJIFILM Wako Pure Chemical Corp., Osaka, Japan) in 0.1 M potassium phosphate buffer (pH 7.8) with 1 mM diethylenetriaminepentaacetic acid (Tokyo Chemical Industry, Tokyo, Japan) at 37 °C for 1 h. NAC polysulfides (final concentration: 1 mM) were then added and incubated at 37 °C for 3 h. After the reaction, excess 2-iminothiolane and NAC polysulfides were removed by a HiTrap Desalting column.

### 4.4. Measuring Polysulfides

The levels of polysulfide were measured following our previous report [26]. In brief, HSA and poly-NAC-Sn-HSA (5 μM) were incubated with L-ascorbic acid (52.8 mg/mL) in 1 M potassium hydroxide at 37 °C for 4 h. Zinc acetate solutions (1%, 600 μL for 200 μL samples) were then added and mixed well, followed by the centrifugation (8000× *g*, 5 min). The supernatants were gently aspirated and then washed three times with 1 mL of water. Following the final step of removing the supernatant, 500 μL of water was added and mixed well. A mixture of 50 μL of 20 mM DPDA in 1.2 N HCl and 50 μL of 30 mM FeCl_3_ in 7.2 N HCl was added to the solution and thoroughly vortexed. The samples were incubated at room temperature for 30 min for coloring. The resulting samples were centrifuged at 8000× *g* for 5 min, and 200 μL of each solution was transferred to 96-well plates for absorbance measurement at 665 nm. A standard curve was generated using Na_2_S (15.6 to 250 μM).

### 4.5. Detection of Reactive Oxygen Species Induced by Myoglobin In Vitro

Pig kidney epithelial cells, LLC-PK1 (ATCC, Manassas, VA, USA) at passage number 194 to 202 were cultured at 37 °C in humidified air under 5% CO_2_ in M199 Medium containing 10% FBS. LLC-PK1 cells were inoculated in a 100 mm^2^ dish in 10 mL medium at a concentration of 10^5^ cells/mL. The medium was changed once in two or three days until the cells get confluence (4–6 days). The cells were seeded in 96 well plates at a concentration of 1.0 × 10^4^ cells and cultured for 24 h. The supernatant was removed and CM-H_2_DCF-DA (final concentration: 5 μM) in DPBS (+) was applied. After incubation for 30 min, the CM-H_2_DCF-DA solution was removed and supersulfide samples (100 μL) were applied for 2 h. The fluorescence excitation at 485 nm and emission at 535 nm was measured on a microplate reader.

### 4.6. Cellular Uptake of Supersulfides in LLC-PK1 Cells

LLC-PK1 cells were seeded in 96 well plates at a density of 1.0 × 10^4^ cells and cultured for 24 h at 37 °C in a 5% CO_2_ humidified incubator. The cells were washed in PBS and supersulfide samples (20 μM in DPBS (+)) were treated. After 2 h of incubation, the cells were washed in PBS and incubated with 5 μM of SSP4 in 1 mM hexadecyltrimethylammonium bromide/PBS for 10 min at 25 °C. The fluorescence intensity (ex. 485 nm/em. 535 nm) was measured.

### 4.7. Glycerol-Induced Acute Kidney Injury Model In Vivo

All animal experiments were approved by the Institutional Animal Care and Use Committees at Tokushima University and Wakayama Medical University. ICR male mice (6 weeks old) were obtained from Japan SLC (Shizuoka, Japan). Mice were provided with a regular diet and water and housed in a temperature-controlled room with a 12-h dark/light cycle. Under the isoflurane anesthesia, 50% of glycerol (10 mL/kg) was injected at one-half of the dose in each hindlimb muscle. Supersulfide samples were treated intravenously right before the glycerol treatment. S_4_-HSA and poly-NAC-S2-HSA were injected at a dose of 1 μmol/kg and NAC-S2 was 100 μmol/kg. Mice were euthanized after 24 h from the administration of glycerol and plasma was collected. The systemic blood vessels were perfused from the heart with PBS, and the kidneys were collected. BUN and serum creatinine were measured using a Urea Nitrogen Colorimetric Detection Kit (Thermo Fisher Scientific, Waltham, MA, USA) and Creatinine Assay Kit (Cayman, Ann Arbor, MI, USA), respectively.

### 4.8. Renal Histology

Kidneys were frozen in an O.C.T. compound (Sakura Finetek Japan, Tokyo, Japan). A 4-μm-thick and 10-μm-thick section was cut for H.E. staining and others. The sections were fixed in 4% paraformaldehyde for 15 min at 25 °C. For the H.E. stain, Mayer’s Hematoxylin solution was applied to the sections for 10 min after PBS wash. Mayer’s Hematoxylin solution (FUJIFILM Wako Pure Chemical Corp.) was then washed out with tap water, and 1% Eosin Y solution (FUJIFILM Wako Pure Chemical Corp.) was applied for 1 min. The sections were dehydrated and cleared using EtOH and xylene. Sections were observed using a BZ-9000 microscope (Keyence, Osaka, Japan). To detect apoptosis in the renal tissue, a TUNEL Assay Kit Edu-Orange (Abcam, Cambridge, UK) was used and observed using a confocal microscope (FV3000, OLYMPUS, Tokyo, Japan). To detect oxidative stress in the renal tissue, immunohistochemistry for 8-OHdG was performed. Sections were blocked with 4% Block ACE (KAC Co., Kyoto, Japan) for 1 h at 25 °C. The primary antibody (8-OHdG Polyclonal Antibody, bs-1278R, BIOSS Antibodies, Beijing, China, diluted 1:250) was exposed for 2 h at 25 °C. After PBS washing, goat anti-rabbit IgG Cy5 (ab6564, Abcam, diluted 1:300) as the second antibody was reacted for 1 h at 25 °C. The sections were washed and mounted using VECTASHIELD mounting medium with DAPI (Vector Laboratories, Newark, CA, USA). Quantitative analysis of the immunohistochemistry and TUNEL stain was performed using Image J software 1.53 k (National Institutes of Health, Bethesda, MD, USA).

### 4.9. Statistical Analysis

All statistical analyses were performed using GraphPad Prism 10.1.0 software (GraphPad Software, Boston, MA, USA). Differences between the groups were evaluated by a one-way ANOVA test with the Turkey multiple comparisons test. All values are represented by the mean ± S.D. A probability value of less than 0.05 was considered to be significant.

## 5. Conclusions

In this study, poly-NAC-Sn-HSA, a novel supersulfide donor containing polysulfides in an oxidized form was developed. Poly-NAC-Sn-HSA decreased the levels of ROS without being taken up in renal tubular cells. On the other hand, S_n_-HSA, a reduced polysulfide donor synthesized by mixing Na_2_S_n_ and HSA, transferred supersulfides into cells and showed antioxidant activity. Poly-NAC-S2-HSA and S_4_-HSA, each of which has four to five supersulfurs per HSA, were selected as representatives of these donors and evaluated for their therapeutic effects in rhabdomyolysis-induced AKI mice. Both donors suppressed the increase in blood parameters such as BUN and creatinine and prevented kidneys from oxidative stress and apoptosis. All those data indicate poly-NAC-S2-HSA and S4-HSA have therapeutic effects on rhabdomyolysis-induced AKI by preventing renal damage from oxidative stress.

## Data Availability

Data is contained within the article.
